# Editorial: *Antibiotics* Special Issue on the Use of Antibiotics in Primary Care

**DOI:** 10.3390/antibiotics10091083

**Published:** 2021-09-08

**Authors:** Gloria Cordoba

**Affiliations:** 1 International Center for Antimicrobial Resistance Solutions (ICARS), 2300 Copenhagen, Denmark; gloriac@sund.ku.dk; 2 Department of Public Health, University of Copenhagen, 1014 Copenhagen, Denmark

For many decades, the importance of increasing knowledge on the rational use of antibiotics has not been given the priority it deserves. Many of us, as health care professionals, were taught in university about the benefits of using antibiotics, without critical argumentation of the other side of the coin, that is, the harms. In recent years, robust evidence has shown that exposure to antibiotics does offer benefits for patients but can also entail adverse effects (e.g., gastrointestinal symptoms, rash) [1] and increases the probability of selection for resistant commensal bacteria at the individual [2] and societal level [3]. Primary care accounts for more than 80% of antibiotic use worldwide. Thus, decreasing the inappropriate use of antibiotics in primary care is crucial not only to slow down the spread and development of antibiotic resistance, but also to deliver high quality health care to patients.

In this Issue, primary care encompasses the wide range of first-contact points within a health care system (i.e., general practice, out-of-hour services (OOH), nursing homes and pharmacies) where patients seek care for the management of community-acquired infections. This Issue showcases evidence that contributes knowledge not only on the drivers and magnitude of the problem (i.e., inappropriate use of antibiotics in primary care) across very heterogeneous countries, but also highlights the importance of using mixed-method approaches to generate evidence on highly effective interventions tailored to the context.

For example, in this Special Issue, different qualitative studies shed light on context-specific factors that need to be taken into consideration to advance the implementation of relevant and effective interventions. Colliers et al. [4] provide a rich understanding of the delivery of OOH services in Belgium; using an innovative qualitative approach (i.e., video elicitation interviews). They show that GPs make assumptions about patients’ reasons for seeking health care without objectifying or verifying such assumptions with the patient. The study clearly shows the need for interventions focusing on improving bi-directional doctor–patient communication. Another key finding is that lack of continuity of care makes it difficult to implement safety-netting strategies such as the wait-and-see strategy in OOH.

Borek et al. [5] in England and Sharaf et al. [6] in Qatar show that despite contextual differences, focusing on the implementation of organizational interventions would likely have a high impact on decreasing the inappropriate prescription of antibiotics and increasing engagement with antimicrobial stewardship initiatives among primary health care professionals.

Poss-Doering et al. [7] describes the German context, reporting on the assessment of implementation outcomes (i.e., acceptability, determinants for uptake) alongside a cluster randomized trial. The highest uptake was reported for educational components such as feedback reports, e-learning modules and disease-specific quality cycles. Uptake was facilitated by the motivation experienced by GPs belonging to primary care networks. They further showed the importance of prioritizing organizational interventions to facilitate the participation of GPs in these networks and to facilitate the economic sustainability of quality cycles.

Finally, Saha et al. [8] report the findings of a qualitative study to explore GPs’ and pharmacists’ interprofessional attitudes and challenges for implementing GP–pharmacist collaborative antimicrobial stewardship activities in Australia. They show that interprofessional education, trust and competencies on antimicrobial stewardship must be improved if this collaborative work is to be successful.

Another methodological approach included in this Issue is a systematic review and meta-analysis on long versus short courses for the management of acute streptococcal pharyngitis [9]. This study goes beyond the assessment of treatment choices (i.e., narrow-spectrum antibiotics should always be the first choice independent of length of treatment) and calls attention to ethical considerations regarding funding priorities. Trials testing the effectiveness of critically important antibiotics should not be carried out in primary care in order to discourage the use of these types of antibiotics at this level.

Another set of evidence relies on observational studies to call attention to specific actions that can decrease the inappropriate use of antibiotics in primary care. Wolterink et al. [10] describe the management of urinary tract infections (UTIs) in elderly men in the Netherlands. Using register-based data, they found that about a quarter of men treated with Nitrofurantoin as the first option of treatment experienced treatment failure. Thus, it would be important to prioritize the implementation of high-quality trials comparing treatment options in elderly men to be able to change treatment choice recommendations in current clinical guidelines.

Rojas et al. [11] describe the Spanish context and its challenges related to enacting policies aimed at reducing the inappropriate use of antibiotics. Using a time series analysis from register-based data, they show that among the following actions—(a) launching of the National Program against Antimicrobial resistance (PRAN), (b) implementation of a co-payment scheme and (c) changes in drug packaging to ensure that packaging was in line with treatment recommendations—only the introduction of the PRAN was associated with a steady reduction in the consumption of antibiotics.

Batenburg et al. [12] illustrates the methodological challenges in quantifying the role of prescribing styles (i.e., the personal tendency to prescribe antibiotics) in the development and spread of AMR at the primary care level. Although the analysis is performed on data from primary care in the Netherlands, the methodological challenges apply to all settings where, despite having an excellent IT infrastructure supporting the surveillance of antimicrobial use and antimicrobial resistance, there are still important gaps regarding the correct processes to carry out surveillance of AMR inprimary care.

Martinez-Gonzalez et al. [13] offer a detailed description of the trends and determinants for consumption of antibiotics in primary care in Switzerland. This observational study demonstrates the importance of implementing interventions addressing organizational factors, such as decreasing workload, if a sustainable decrease of unnecessary prescription of antibiotics in primary care is to be achieved within the Swiss context.

Siltrakool et al. [14] describe the role of community pharmacists within primary care in Thailand and demonstrate that outdated guidelines are creating a bottleneck in the achievement of evidence-based practice by Thai pharmacists. Thus, updating the clinical guidelines in Thailand should be a priority to decrease the inappropriate use of antibiotics and secure effective partnerships between clinicians and pharmacists.

Finally, Sommer-Larsen et al. [15] describe the Danish context regarding the diagnosis and management of suspected urinary tract infections (UTIs) in nursing home residents. It demonstrates that even in countries with a decreasing trend in the use of antibiotics in primary care, there are still areas for improvement, such as implementing interventions to improve the quality of diagnostic processes and treatment decisions in elderly patients with suspected UTIs.

Overall, the inappropriate use of antibiotics in primary care will not be resolved if we do not take a system-based approach and acknowledge the importance of context-tailored interventions. It is paramount to extend beyond individual behaviors by setting up multifaceted interventions that target changes at the organizational, prescriber and individual levels.
